# Sustainable Concrete Using Ceramic Tile Waste as a Substitute for Brick Aggregate

**DOI:** 10.3390/ma18133093

**Published:** 2025-06-30

**Authors:** Kamal Hosen, Alina Bărbulescu

**Affiliations:** 1 School of Ocean and Civil Engineering, Shanghai Jiao Tong University (SJTU), 800 Dong Chuan Road, Minhang District, Shanghai 200240, China; kamalcivilsub@gmail.com; 2 Department of Civil Engineering, Transilvania University of Brașov, 5 Turnului Str., 500152 Brașov, Romania

**Keywords:** sustainable concrete, ceramic tile waste, brick aggregate replacement, concrete recycling, concrete performance

## Abstract

Recycled materials have gained extensive recognition in many industrial sectors for enhancing sustainability and reducing environmental impacts. Combining ceramic tile waste (CTW) in concrete mixes with recycled aggregate will help lower natural aggregate demand and reduce the amount sent to landfill. This paper aims to study the mechanical properties of CTW in concrete mixes as a brick aggregate replacement and its impact on concrete strength and durability. To evaluate and assess their strength and durability, three types of concrete cubes were prepared using 20%, 40%, and 70% of waste ceramic tiles as a replacement for coarse aggregate. Two kinds of concrete samples were also prepared with conventional coarse aggregate as the control specimen (CC). A 1:2:4 concrete mixed ratio was used in this research with a 0.50 water–cement ratio. The samples were tested after 14 days and 28 days to assess their mechanical properties, including strength and durability. When CTW was added to concrete mixtures instead of brick chips, the mechanical strength rose considerably, and the water absorption performance increased. Moreover, replacing brick chips with ceramic waste in concrete could have significant environmental benefits.

## 1. Introduction

With over 35% of global CO_2_ emissions and more than 40% of total energy use attributed to it, the construction industry stands as one of the most resource- and emission-intensive sectors worldwide [1,2]. The widespread acceptance of concrete as a popular building material is primarily due to its accessibility, cost-effectiveness, functionality, and ease of use [3,4]. Every year, roughly 30 billion tons of concrete are produced globally, contributing to about 3% of the world’s energy consumption and approximately 8% of total greenhouse gas emissions [5]. The aggregate forms the more significant part of concrete, ranging from 60% to 70%, and is mainly made from natural and nonrenewable materials [6]. Recycling coarse aggregates into regular concrete production in the cement industry has recently gained significance as part of the drive for circularity [7,8]. This trend has occurred primarily due to the need for a solution to reducing environmental impacts and the exhaustion of resources as natural coarse aggregates have increasingly taken the spotlight [9].

In 2020, around 26 Gt of concrete was consumed globally, corresponding to some 20 Gt of aggregates [10]. This immense demand, therefore, makes a strong case for developing sustainable means of concrete production. However, this transition carries a considerable number of challenges because of the variability in the quality of recycled concrete aggregate (RCA) [11]. The production of ceramic tiles has doubled in the last few years, going from 8581 million m^2^ produced around the globe in 2009 to 16,093 million m^2^ in 2020 [12]. Spain is the top producer of ceramic tiles in the European Union and the second largest worldwide, with 415 million m^2^ of ceramic tiles exported in 2020, representing 81.4% of the total production. The reuse of CTW contributes to circular economy strategies by decreasing reliance on virgin raw materials and mitigating the environmental impacts associated with landfill disposal [13].

Construction and demolition activities occur globally, accounting for more than 75% of total waste generation [14]. In addition, ceramic materials comprise approximately 54% of the waste from construction and demolition works [15]. Because ceramics are brittle and easily break during production, transport, and installation, their use in building and structural construction is growing [16]. Examples include tiles, electrical insulators, sanitary fittings, and other ceramic objects. Roughly 75% of all garbage produced worldwide results from construction and demolition materials [17].

An advantageous type of recycling is collecting waste materials as raw inputs for producing corresponding virgin products [18]. Given that CTW has the potential for sustainable construction material recycling, its integration into ceramic tiles has gained popularity. For example, CTW is utilized for paving bricks, porcelain tiles, un-few fired bricks, and foam ceramics [18]. CTW has excellent potential to be used as a sustainable construction material in producing Ultra-High-Performance Concrete (UHPC) together with seawater concrete and additional construction materials [19]. Implementing CTW materials in construction works would drive urban sustainability while decreasing waste production and directly help achieve targets under SDGs 11 and 12 from the United Nations [20].

In their experiments, Poon and Chan [21] replaced 20% of natural sand with finely crushed brick or tile waste in concrete and compared conventional vs. double mixing methods. This replacement slightly reduced the density of the concrete (e.g., control: 2390 kg/m^3^, brick mix: 2340 kg/m^3^, tile mix: 2370 kg/m^3^). The double mixing method increased compressive strength, with tile aggregate concrete achieving 55.3 MPa at 28 days—21.5% higher than conventional mixing. All mixes met the 45 MPa target strength, and drying shrinkage stayed under 0.075%, showing they are suitable for general applications. Tanash et al. [22], exploring the use of ceramic waste, found that concrete with coarse ceramic aggregate had a higher compressive strength than the control, and its strength increased beyond 28 days. Elçi [23] investigated employing 100% crushed wall and floor tile waste as fine and coarse aggregate replacements in concrete. The study compared their performance to a conventional limestone aggregate. Concrete made with wall tile aggregate (WTA) showed a significantly lower performance, with a fresh density 20% lower (1670 kg/m^3^ vs. 2377 kg/m^3^) and splitting tensile strength reduced by 15.6% (2.70 MPa vs. 3.20 MPa) at 28 days. In contrast, floor tile concrete (FTC) performed closer to limestone concrete (LSC), with only 14% lower fresh density (2036 kg/m^3^) and slightly higher splitting tensile strength (3.31 MPa vs. 3.20 MPa). While tile aggregates had a higher water absorption capacity (WTA: 18.57%, FTC: 12.46%, LSC: 6.06%), FTC offered a comparable mechanical strength, suggesting its potential as a sustainable aggregate alternative.

Medeiros et al. [24] studied eco-friendly concrete made using porcelain polishing and scheelite wastes, performing microstructural analysis to see how these materials impact concrete’s properties. Their research contributes to a broader understanding of how various waste materials can be integrated into concrete formulations to improve sustainability. Koushkbaghi et al. [25] investigated the incorporation of RCA and rice husk ash (RHA) in concrete mixtures. When RCA entirely replaced the natural aggregate, the splitting tensile strength decreased by 22.2% in mixes without fibers and by 14.7% in those containing fibers. In fibrous mixes containing 20% RHA, tensile strength experienced only slight reductions of 2.2% at 28 days and 5.6% at 90 days, highlighting the positive effect of RHA on the fiber–matrix interface.

Keshavarz and Mostofinejad [26] conducted an experimental study on the use of porcelain and red ceramic waste as partial replacements for coarse aggregates in concrete. A total of 65 specimens were tested for compressive, tensile, and flexural strength, as well as water absorption. The results showed that porcelain significantly enhanced the concrete’s mechanical properties, improving compressive strength by 41% and flexural strength by 67%. However, both porcelain and red ceramic increased water absorption (54% for porcelain aggregate concrete, compared to 91% for brick aggregate concrete).

Juan-Valdés et al. [27] evaluated concrete mixes where natural coarse aggregates were partially replaced with recycled ceramic aggregates. The study included a control mix (using only natural aggregates) and a recycled mix (RC-RA). Mechanical strength, density, porosity, and microstructural analysis revealed that the recycled concrete achieved compressive strengths at 28 days equal to or exceeding those of conventional concrete. Additionally, it exhibited a more refined pore structure, confirming its potential as a sustainable construction material.

Mousavi et al. [28] examined the use of recycled ceramic aggregates from waste tiles and sanitary ware (CS) as partial replacements for natural coarse aggregates in high-strength concrete (HSC). Seven mixes with replacement levels ranging from 0 to 30% were tested for slump, compressive strength, water absorption, and chloride penetration. The study found that 20% CTW replacement improved mechanical strength, while 10% CS replacement enhanced durability. However, higher replacement levels led to increased water absorption and chloride ingress. The authors concluded that ceramic waste can be effectively and sustainably used to produce HSC (≥60.7 MPa), with optimal performance at 10–20% replacement—offering environmental benefits without compromising structural integrity.

Although CTW has been widely studied as a sustainable material in concrete, most research to date has focused on its use as a fine aggregate or at low replacement levels for natural coarse aggregates [21,23,29]. There is limited investigation into the use of CTW as a full or partial substitute for brick chips, which are commonly used as coarse aggregates in many developing countries. This gap is significant, as brick chips possess distinct physical properties—such as a higher porosity and lower density compared to natural stone—which may influence performance outcomes. Moreover, high replacement levels of CTW (above 40% by mass) remain underexplored, and a few existing studies report inconsistent results regarding strength and durability performance [23,29]. Direct comparative analyses between CTW and brick aggregate concrete are particularly scarce. While some studies have examined basic durability indicators like water absorption [23], more comprehensive assessments of long-term performance—including shrinkage, freeze–thaw resistance, and sulfate attack—are lacking. Similarly, despite frequent references to the environmental benefits of CTW, quantitative evaluations such as cost–benefit analyses and environmental impact assessments are still rare [30]. These research gaps limit a full understanding of CTW’s potential as a sustainable coarse aggregate, particularly in contexts where brick aggregate concrete is prevalent.

Ceramic tile waste (CTW), particularly porcelain based, exhibits superior physical and chemical properties compared to other recycled aggregates such as crushed concrete or red ceramic. These advantages include higher particle density, lower porosity, and a more uniform mineral composition [23,26,30], all of which contribute to improved mechanical strength and durability when incorporated into concrete. These benefits have been confirmed in recent experimental studies [23,26]. Building upon this prior research, the present study demonstrates that CTW concrete—particularly at a 40% replacement level—outperforms conventional brick aggregate concrete in terms of mechanical performance.

This study aimed to address several of the previously identified research gaps by evaluating the effects of incorporating CTW as a partial replacement for brick chips in concrete at replacement levels of 20%, 40%, and 70%—higher than those commonly reported in the literature. The focus on brick chips, rather than natural coarse aggregates, reflects construction practices in many developing regions, where brick aggregate is widely used. This enhances the practical relevance and applicability of the findings.

A series of experimental tests were conducted to assess key concrete properties, including compressive strength, split tensile strength, unit weight, water absorption, and modulus of elasticity, to better understand the performance of CTW as a coarse aggregate.

The results of this study show that moderate replacement levels, particularly at 40%, enhanced both the strength and durability of the concrete compared to the control mix containing only brick aggregate. At higher replacement levels (e.g., 70%), performance remained comparable to traditional brick aggregate concrete.

In addition, the study highlights the sustainability benefits of using CTW, such as reducing construction waste sent to landfills and conserving natural resources. These findings offer valuable insights into the potential of CTW as a sustainable and effective alternative to traditional coarse aggregates, particularly in regions where brick-based concrete is widely used.

## 2. Materials and Methods

### 2.1. Materials

The following materials were used to cast the study samples.

*Ordinary Portland cement* that adhered to the relevant Chinese GB standards, including GB 50010 [31], GB 50086 [32], GB/T 14684 [33], and GB/T 3186 [34].

- *Fine aggregate*: The sand that served as the fine aggregate in cement was taken from Jiangsu province.
- *Coarse aggregate*: Brick chips (brick gravel and brick ballast) are a type of coarse aggregate made up of bricks. Typically, 19 mm or 20 mm degraded brick chips are utilized as coarse material for this operation. Brick chips were partially replaced in this work by porcelain floor tiles. Ceramic tile waste (CTW) was gathered in Shanghai and was carefully divided into uniform aggregate sizes. They primarily consisted of a mixture of natural raw materials, including approximately 55–60% silica (SiO_2_), 25–30% alumina (Al_2_O_3_), along with smaller amounts of feldspar and other mineral oxides. This composition is typical for porcelain ceramics and is achieved through a high-temperature firing process (1200–1400 °C), which produces a dense and vitrified material. The high silica and alumina content contributes to the tiles’ low porosity and high mechanical strength, directly influencing the concrete’s overall durability and performance when these waste tiles are used as partial aggregate replacements. Additional components for concrete, like water, additives, aggregate, and cement, were gathered or handled locally. The parameters of the aggregates employed in this study are displayed in Table 1.

Four mixes, including the control mix (CC), were made. A 1:2:4 ratio represents the combination of cement, fine aggregate, and coarse aggregate. The designated formula determined the amount of the materials for each mix, including the control mix, when using a constant 0.50 water-cement ratio. All mixes were made with a 1% (weight of cement) super-plasticizer additive to create high-workability concrete without sacrificing strength. Table 2 shows the concrete mixing ratios.

**Table 1:** Aggregate properties.

Property	Fine Aggregate (Sand)	Coarse Aggregate (Brick Chips)	Ceramic Tile Aggregate
Fineness Modulus	3.15	6.15	6.90
Maximum Aggregate Size (mm)		20	20
Unit Weight (kg/m^3^)	1700	1125	1390
Voids (%)	35.10	55.02	41.10
Specific gravity	2.60	1.75	2.30
Water absorption (%)	1.00	16.0	1.20

Three levels of CTW replacement (20%, 40%, and 70%) were selected to represent low, intermediate, and high levels of substitution. The 20% replacement level aimed to assess whether CTW could be integrated into concrete without significantly affecting its mechanical properties compared to conventional brick aggregate mixes. The 40% level was chosen as an intermediate value, informed by previous studies [23,29], where improvements in both strength and durability were anticipated. The 70% replacement level was included to explore the upper threshold of CTW incorporation and to evaluate how high levels of substitution influence the structural performance and durability of the concrete.

### 2.2. Tests

Concrete workability is described as the ease with which freshly mixed concrete can be handled, placed, and compacted without loss of homogeneity [35]. In this study, the workability of each concrete mix was assessed using the ASTM C143 standard [36].

Cubs with a cross-section of 150 mm × 150 mm were cast and subjected to mechanical testing. For each kind of material, we cast 25 cubes, of which 24 were used for the experiments that were performed in triplicate. The samples were cured and tested after 14 days and 28 days.

The unit weight, compressive strength, and split tensile tests were performed at 14 and 28 days, whereas modulus of elasticity and water absorption tests were performed only at 28 days. The compressive strength was performed according to the standard for mechanical strength testing of concrete, including concrete with CTW, which is ASTM C39 [37]. For the split tensile test, we followed the prescriptions from the standard ASTM C496 [38,39].

The mechanical tests on the concrete specimens were performed using a Universal Testing Machine (UTM) supplied by Zhejiang Chenxin Machinery Equipment Co., Ltd., Shangyu, China.

## 3. Results and Discussion

### 3.1. Workability

After testing it was found that standard brick aggregate concrete had a slump value of 80 mm. The slump value rose when brick chips were substituted with ceramic waste tiles. They were 90, 100, and 110 for CWA-20, CWA-40, and CC-70, respectively. These results can be explained by the fact that the flat surface of ceramic tile waste allows particles to flow more efficiently and with less resistance.

Our results are in line with other previous works. For example, Paul et al. [29] observed an increase of 31%, 52%, and 103% in slump values with respect to the control mix at 20%, 50%, and complete replacement of the coarse aggregates with waste ceramic tile aggregates. Ambrose et al. [40] observed a sharp slump reduction from 158 mm to 3 mm at complete sand replacement, citing ceramic fines’ porous and angular nature. Awoyera et al. [30] reported a decrease to 40 mm at complete coarse aggregate replacement, attributing it to the ceramic particles glazed and irregular surfaces. The improved slump in this study is due to the smoother texture and lower water absorption of ceramic tiles compared to brick chips, which reduces internal friction and enhances flowability.

### 3.2. Unit Weight Test Results

**Figure 1:**
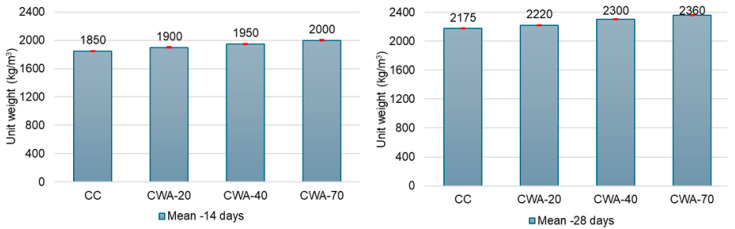
Unit weight test comparison at 14 days (**left**) and 28 days (**right**).

Concrete density determines the unit weight of concrete, which serves two vital purposes: structural assessment, material design, and cost management. Various mixed proportions with unit weight calculations for varying curing times are used in this study. Figure 1 illustrates how a more significant percentage replacement of ceramic waste aggregate may result in a higher unit weight due to the lower void content and higher specific gravity of the waste aggregate.

After 14 days, the unit weights for CC, CWA-20, CWA-40, and CWA-70 were 1850 ± 3.74 kg/m^3^, 1900 ± 4.97 kg/m^3^, 1950 ± 3.56 kg/m^3^, and 2000 ± 6.68 kg/m^3^, respectively. After 28 days, they increased to 2175 ± 2.16 kg/m^3^, 2220 ± 5.72 kg/m^3^, 2300 ± 3.74 kg/m^3^, and 2360 ± 4.32 kg/m^3^, respectively, for the same materials (Figure 1). The standard deviations are represented by the intervals with close caps, in red, at the top of the bars in Figure 1.

This increase is by about the same value (around 320 kg/m^3^) and is due to the higher specific gravity (2.30) and lower void content (41.10%) of the ceramic tile aggregate compared to the brick chips (specific gravity 1.75 and voids 55.02%).

Keshavarz and Mostofinejad [26] found that concrete made with porcelain tile waste as the coarse aggregate had a higher unit weight because porcelain has a greater density and less porosity than red ceramic (brick) waste. Elçi [23] reported a notable drop in fresh and hardened concrete densities when crushed ceramic tiles entirely replaced aggregates, with hardened densities decreasing to around 1670 kg/m^3^. This was due to the ceramic aggregates’ higher porosity and lower specific gravity. Similarly, Ambrose et al. [40] okfound that fresh concrete density gradually decreased from about 2616 kg/m^3^ to 2471 kg/m^3^ as ceramic tile waste replaced natural sand, caused by the recycled ceramic’s lower specific gravity and higher void content.

The higher unit weight in this study is due to the denser packing of CTW. Ceramic tiles also have a higher specific gravity than brick chips, which have lower density. Together, these factors create a more compact concrete matrix. This denser matrix supports the improved mechanical properties observed.

### 3.3. Compressive Strenght Test Results

Testing the compressive strength of concrete is important because it measures the material’s capacity to withstand axial loads, helping to assess its structural performance and durability over time [41,42]. Structures made from concrete with a high compressive strength have better capability to handle larger loads while decreasing their risk of breaking and other types of strength-reducing damage.

**Figure 2:**
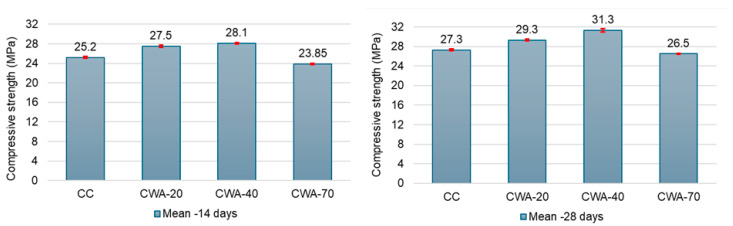
Comparison of the 14-day (**left**) and 28-day (**right**) compressive strength between CC and CWA. The standard deviations are represented by the intervals with close caps, in red, at the top of the bars.

Each concrete mixture showed an augmentation in compressive strength among both concrete mixtures comprising ceramic waste aggregates together with conventional brick aggregates as the specimens aged. After 14 days, the compressive strength for CC, CWA-20, CWA-40, and CWA-70 were 25.2 ± 0.22 MPa, 27.5 ± 0.22 MPa, 28.1 ± 0.14 MPa, and 23.85 ± 0.15 MPa, respectively. They slowly increased reaching 27.3 ± 0.16 MPa, 29.3 ± 0.22 MPa, 31.3 ± 0.36 MPa, and 26.45 ± 0.14 MPa, respectively (Figure 2).

The compressive strength improves with the addition of ceramic tile waste, peaking at a 40% replacement level, achieving 28.1 MPa at 14 days and 31.3 MPa at 28 days. These values are about 15.66% and 16.61% higher than those of the control mix. At 70% replacement, the strength drops slightly but remains comparable to that of CC, showing that moderate amounts of ceramic waste improve concrete strength. The high-rated compressive strength of the CWA can be attributed to specific reasons: its composition is more uniform, and the way it bonds and interlocks in the concrete mix is enhanced.

Our results are in concordance with those in previous studies, as follows. Awoyera et al. [30] documented significant strength gains when ceramic waste replaced natural aggregates, attributing the improvement to ceramic particles’ angularity and rough texture, which improved the interfacial bond. Paul et al. [29] reported that compressive strength decreases with increasing waste ceramic tile aggregate content, showing about a 12% reduction at 10% replacement and up to 52% reduction at 100% replacement after 28 days of curing. Ambrose et al. [40] observed progressive strength increases up to the complete replacement of natural sand with recycled ceramic tile fines, suggesting pozzolanic activity and improved particle packing as contributing factors. This improvement is due to the ceramic tiles’ dense and uniform microstructure, which enhances bonding within the concrete and reduces porosity.

The reduction in compressive strength observed at the 70% CTW replacement level is primarily attributed to weaker bonding at the interfacial transition zone between the aggregate and the cement paste matrix. Ceramic tile waste possesses a denser texture and smoother surface compared to brick chips, resulting in lower surface roughness. This diminished roughness reduces mechanical interlocking and weakens paste adhesion, ultimately decreasing the effectiveness of stress transfer between the aggregate and the cement matrix. Additionally, while increased CTW content may appear to improve workability, the smoother ceramic surface disrupts aggregate packing and alters the internal gradation, which negatively impacts cohesion and overall density at high replacement levels. These combined factors contribute to the observed decline in compressive strength at 70% CTW replacement.

### 3.4. Modulus of Elasticity Test Results

The modulus of elasticity of concrete indicates the material’s stiffness, reflecting the extent to which it can elastically deform under applied stress. This test is essential because it helps predict concrete behavior under load, especially in terms of deformation and structural stability, which is necessary to design safe and efficient structures.

**Figure 3:**
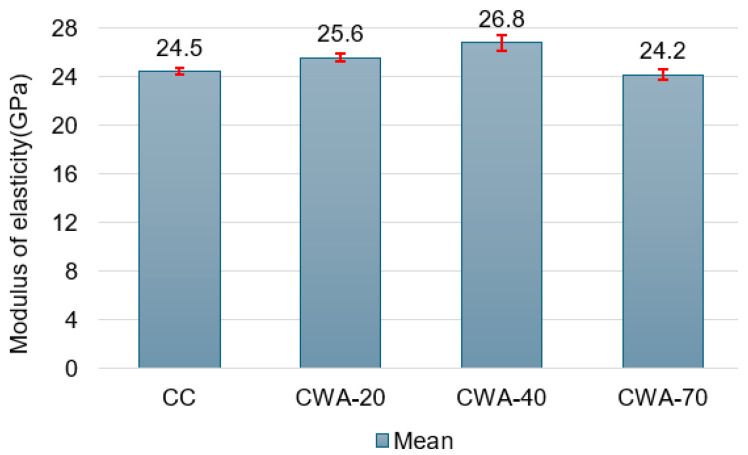
Modulus of elasticity at 28 days. The standard deviations are represented by the intervals with close caps, in red, at the top of the bars.

Incorporating ceramic waste material into concrete results in variation in the modulus of elasticity (determined at 28 days), from 24.5 ± 0.29 GPa for CC to 25.6 ± 0.36 GPa for CWA-20, reaching its maximum of 26.8 ± 0.65 GPa for CWA-40, and then decreasing to 24.2 ± 0.46 GPa for CWA-70 (Figure 3).

The highest recorded value was about 8.10% higher than that for CC. The minimum modulus of elasticity was 0.3 GPa lower than that of the control concrete, so it remained close to the control value. The results indicate that introducing a moderate ceramic waste percentage in the composition improves the concrete’s elastic behavior. Our result is better than the findings of Awoyera et al. [30], who reported enhanced stiffness with ceramic aggregate incorporation, albeit with a minor increase of approximately 4%. So, the concrete became stiffer and more resilient to deformation as ceramic waste was added due to the ceramic aggregates’ higher stiffness and better packing. 

### 3.5. Split Tensile Strength Test Results

The split tensile strength test involves applying a compressive load along the diameter of a cylindrical concrete specimen, inducing indirect tensile stresses perpendicular to the applied load. The load is gradually increased until the specimen fails. This test is essential for evaluating concrete’s resistance to tensile cracking, which is critical for understanding the material’s performance and durability under service conditions. While the standard practice uses cylindrical specimens for this test, several researchers [43,44] have also used cube specimens in experimental studies, as was carried out in this study.

**Figure 4:**
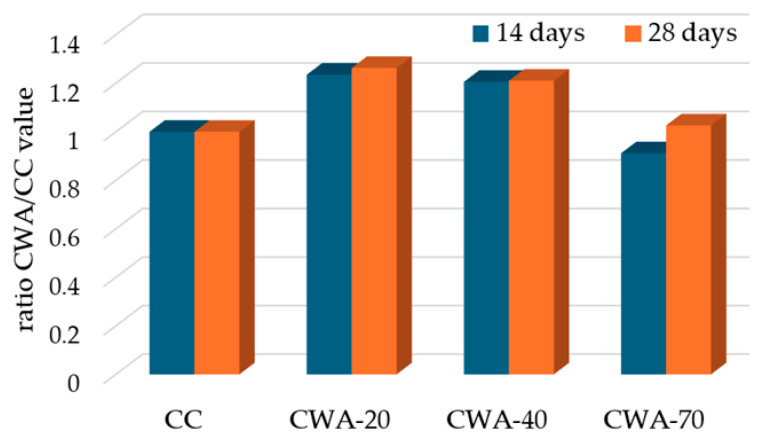
Comparison of the split tensile strength of conventional concrete CC and CWA concrete at 14 days and 28 days—represented as ratios CWA value/CC value at 14 days (blue) and 28 days (orange).

In our experiments, the split tensile strength results after 14 days were 3.4 ± 0.11 MPa, 4.2 ± 0.18 MPa, 4.1 ± 0.12 MPa, and 3.1 ± 0.18 MPa for CC, CWA-20, CWA-40, and CWA-70, respectively. After 28 days, the corresponding values increased to 3.8 ± 0.08 MPa, 4.8 ± 0.12 MPa, 4.6 ± 0.11 MPa, and 3.9 ± 0.09 MPa. These results indicate a consistent increase in tensile strength with curing age, demonstrating improved cohesion and hydration in the mixes over time. Using the control concrete (CC) as a reference, the relative ratios of tensile strength for the ceramic waste concrete mixes at both ages are presented in Figure 4.

The split tensile strength of concrete improved when CTW replaced part of the aggregate, reaching a maximum for CWA-20, representing an increase of 23.53% at 14 days and 26.32% at 28 days. For CWA-40, the augmentation with respect to CC was with 20.59%, and 21.05%, respectively. For CWA-70, there was a decrease of 8.8% at 14 days and a slight increase of 2.63% at 28 days by comparison with CC. Other authors similarly documented enhanced tensile strength with ceramic aggregate incorporation, particularly at around 30% replacement [30] and 41% [26]. These results are attributable to the greater density of ceramic tiles and reduced porosity compared to red ceramic waste. They suggest incorporating ceramic waste improves the concrete’s ability to resist tensile stress and cracking, particularly at lower replacement levels.

### 3.6. Water Absorption Test Results

**Figure 5:**
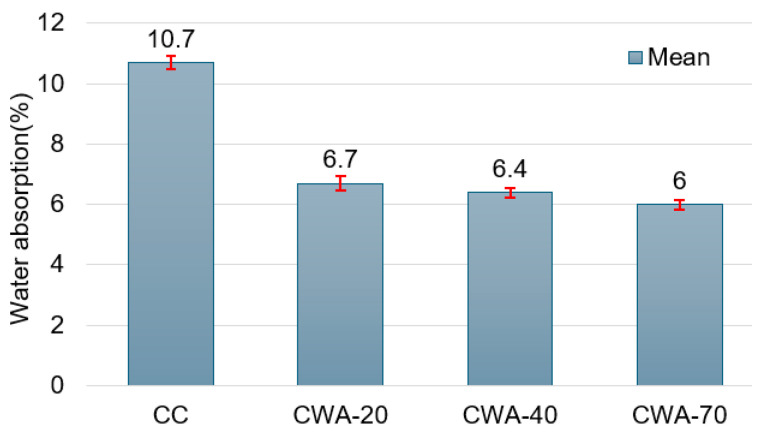
Water absorption of CC and CWA concrete at 28 days. The red vertical lines with caps represent the standard deviation from the mean.

The water absorption test measures the capacity of concrete to absorb water after being immersed for 24 h, offering valuable information about the material’s porosity and permeability. This test is crucial because water absorption directly impacts the durability of concrete. Higher absorption rates (%) typically result in reduced strength and increased vulnerability to environmental damage. In this study, the water absorption rates at 28 days were 10.7, 6.7, 6.4, and 6.00 for CC, CWA-20, CWA-40, and CWA-70, respectively (Figure 5). The higher value was recorded for CC, indicating that the brick chips absorb more water than waste from ceramic tiles. A higher percentage of CWA will induce a reducer porosity and greater concrete density, reducing water absorption.

Ambrose et al. [40] also reported a decrement in water absorption when increasing the ceramic tile content with up to 30% of the fine aggregate. The lowest absorption was around 8% in their study. Our results differ from those of Elçi [23], who indicated that the floor tiles aggregates and limestone aggregates have similar properties, and those of de Brito et al. [45], who found a higher absorption for the material made using ceramic tiles.

### 3.7. Statistical Analysis of the Results

To determine if there is a significant difference between the properties of the four types of materials, we performed non-parametric ANOVA for each type of experiment performed at 14 and 28 days, at a significance level of 5%. The *p*-values are reported in Table 3.

Since all *p*-values are less than the significance level, the null hypothesis that there is no significant difference between the properties of the four types of materials can be rejected in each test.

## 4. Conclusions

In this study, concrete was produced using varying proportions of ceramic tile waste instead of coarse aggregate. To evaluate the structural performance, various tests were performed. The key findings are as follows:

- The slump values for concrete made with CWA ranged from 80 to 110 mm, indicating that a higher ceramic waste content enhances the workability of the mix. Compared to traditional brick aggregate concrete, CWA concrete demonstrated higher unit weight due to its greater specific gravity and reduced porosity.
- Among the different mixes, CWA-40 achieved optimal compressive strength after 14 days of curing, and a 15.66% improvement over conventional brick aggregate concrete, after 28 days.
- The highest modulus of elasticity was observed at CWA-40 after 28 days, representing an 8.10% increase over traditional concrete, CC.
- The split tensile strength peaked at 4.20 MPa and 4.80 MPa with CWA-20 after 14 and 28 days of curing, respectively.

Based on the overall test results, a 40% replacement of brick chips with CTW is recommended to achieve improved strength and durability in concrete. However, to fully assess the potential benefits and limitations of using CTW, further research should be conducted using ceramic tiles of varying compositions. This will allow for a more comprehensive evaluation of the mechanical properties and long-term durability of CTW-incorporated concrete across different material types.

We should also note that incorporating CTW into concrete presents a viable and sustainable solution for construction applications, particularly in industrial settings. CTW concrete demonstrates adequate mechanical strength and durability, making it suitable for pavements, industrial flooring, non-structural elements, precast components, and road sub-base layers [12,29]. Its performance and the environmental benefits of reducing ceramic waste and conserving natural aggregates support its adoption in eco-friendly construction practices. These findings reinforce the potential of CTW concrete as a practical material for industrial use and promote further research into optimizing its composition for broader structural applications.

## Figures and Tables

**Table 2:** Mixing ratio of concrete (1:2:4).

Mix	Ceramic Tiles Replacement (%)	W/C Ratio	Cement (kg/m^3^)	Water (kg/m^3^)	Fine Aggregate (Brick Chips) (kg/m^3^)	Coarse Aggregate (Brick Chips) (kg/m^3^)	Ceramic Tiles Aggregate (kg/m^3^)
CC	0	0.50	403	200	806	1612	0
CWA-20	20	0.50	403	200	806	1289.6	322.4
CWA-40	40	0.50	403	200	806	475.6	644.8
CWA-70	70	0.50	403	200	806	1128.4	483.6

**Table 3:** *p*-values in the ANOVA test.

Test	14 Days	28 Days
Unit Weight	8.28 $\times \;10^{-9}$	3.12 ${\times \;10}^{-5}$
Compressive Strength	3.99 $\times \;10^{-8}$	1.93 $\times \;10^{-6}$
Modulus of Elasticity	-	5.76 $\times \;10^{-4}$
Split Tensile Strength	1.91 $\times \;10^{-4}$	1.96 ${\times \;10}^{-5}$
Water Absorption	-	2.58 $\times \;10^{-8}$

## Data Availability

The original contributions presented in this study are included in the article. Further inquiries can be directed at the corresponding author.

## Abbreviations

The following abbreviations are used in this manuscript:

CTW	ceramic tile waste
CS	sanitary wares
FTC	floor tile concrete
HSC	high-strength concrete
LSC	limestone concrete
RCA	recycled concrete aggregate
RHA	rice hush concrete
UHPC	ultra-high-performance concrete
WTA	wall tile aggregate
